# Implications of Hydrogen Sulfide in Glucose Regulation: How H_2_S Can Alter Glucose Homeostasis through Metabolic Hormones

**DOI:** 10.1155/2016/3285074

**Published:** 2016-07-11

**Authors:** Jennifer Pichette, Jeffrey Gagnon

**Affiliations:** Laurentian University Department of Biology, 935 Ramsey Lake Road, Sudbury, ON, Canada P3E 2C6

## Abstract

Diabetes and its comorbidities continue to be a major health problem worldwide. Understanding the precise mechanisms that control glucose homeostasis and their dysregulation during diabetes are a major research focus. Hydrogen sulfide (H_2_S) has emerged as an important regulator of glucose homeostasis. This is achieved through its production and action in several metabolic and hormone producing organs including the pancreas, liver, and adipose. Of importance, H_2_S production and signaling in these tissues are altered during both type 1 and type 2 diabetes mellitus. This review first examines how H_2_S is produced both endogenously and by gastrointestinal microbes, with a particular focus on the altered production that occurs during obesity and diabetes. Next, the action of H_2_S on the metabolic organs with key roles in glucose homeostasis, with a particular focus on insulin, is described. Recent work has also suggested that the effects of H_2_S on glucose homeostasis goes beyond its role in insulin secretion. Several studies have demonstrated important roles for H_2_S in hepatic glucose output and adipose glucose uptake. The mechanism of H_2_S action on these metabolic organs is described. In the final part of this review, future directions examining the roles of H_2_S in other metabolic and glucoregulatory hormone secreting tissues are proposed.

## 1. Introduction

Hydrogen sulfide (H_2_S) is a colorless and odorless gas that is produced both endogenously by a variety of mammalian cells and by the sulfate reducing bacteria in the lower gastrointestinal (GI) tract. H_2_S has emerged as an important gasotransmitter that regulates several systems including the cardiovascular, GI, immune, endocrine, and nervous systems (reviewed in detail in [1]). One area of recent interest is the potential role that H_2_S may play in glucose regulation and metabolic health. Indeed, several groups have demonstrated that obese and diabetic individuals have altered H_2_S levels in their circulation [2, 3] and tissues [4, 5]. The precise mechanisms of how H_2_S can drive metabolic changes are beginning to be understood. A major factor in the regulation of glucose metabolism is the secretion and action of metabolic hormones. These hormones include insulin, glucagon, leptin, and glucagon like peptide-1. Several groups have already described the action of H_2_S on insulin secretion [6–8]. Furthermore, recent work has demonstrated the effects of H_2_S on downstream hormone signaling [9]. These studies and others suggest that H_2_S may be a potential target in the treatment of metabolic diseases through modulating metabolic hormone secretion and signaling. The goal of this review is to describe the roles of H_2_S in the regulation of metabolic hormone secretion, with a particular focus on insulin, and the downstream signaling of these hormones in the regulation of energy homeostasis.

## 2. H_**2**_S Production

Although the presence of H_2_S in the body has been known for some time, the precise locations of its production remain an active area of research. H_2_S is produced by a large variety of cell types in the body (here named endogenous) and by host microbes including the sulfate reducing bacteria in the GI tract. The main enzymatic machineries in the endogenous production of H_2_S are the cystathionine metabolizing cystathionine-*β*-synthase (CBS) [10] and cystathionine *γ*-lyase (CSE) [11]. Other enzymes such as 3-mercaptopyruvate sulfurtransferase (MST) and cysteine aminotransferase (CAT) are also important in specific tissue types [12]. CSE activity is much higher than CBS in peripheral tissues, while CBS mainly predominates in the brain [13, 14]. The precise mechanisms involving the production of endogenous H_2_S are thoroughly reviewed by Wang in [1]. Once H_2_S is produced in the cell, it can act on different cellular pathways or be stored for later release. H_2_S can store its sulfur group with iron (acid labile sulfur) [15] or in sulfane sulfur (a persulfide) [16] in mammalian tissues. When required and under the appropriate conditions, this bound sulfur can be released as S^2−^, HS^−^, or H_2_S [17].

In addition to endogenous generation, H_2_S can be produced from microorganisms in the GI tract. The gut microbiota aids in the decomposition and harvest of nutrients from food, a crucial step in energy production. Primary fermenters break down protein and complex carbohydrates into short-chain fatty acids (e.g., acetate, propionate, and butyrate) that are an important energy source, and gases (e.g., hydrogen, carbon dioxide) that are released or absorbed by the system. Hydrogenotrophs, or H_2_-consuming bacteria, are essential in keeping luminal hydrogen levels low and stabilizing the environment for these primary fermenters. Among the groups of hydrogenotrophs are methanogens (producing methane), acetogens (producing acetate), and sulfate reducing bacteria (producing H_2_S). Sulfate reducing bacteria use hydrogen or organic compounds as electron donors and use sulfate as their terminal electron acceptor leading to a large production of H_2_S. This process is known as dissimilatory sulfate reduction and can lead to mM concentrations of H_2_S in the lumen [18]. Sulfur sources from diet can originate from amino acids, preservatives, and food additives (carrageenan) or as dietary supplements (chondroitin sulfate) [18]. Microbial produced H_2_S is a significant contributor to the bodies H_2_S pool, as germ free mice have between 50 and 80% less H_2_S in their tissues and circulation [19]. Microbial H_2_S has been associated with both maintaining gastric health and being implicated in disease. Several groups have shown that H_2_S regulates various physiological functions including maintenance of GI barrier function and injury repair [20]. Some earlier studies have suggested that H_2_S may be involved in the etiology of ulcerative colitis [21]. However, more recent work points towards a protective role [22]. Regardless of its source, H_2_S has emerged as a regulator of glucose metabolism. The mechanisms of this action are described below.

## 3. Importance of H_**2**_S in Diabetes and Insulin Regulation

Insulin is one of the most researched and clinically important metabolic hormones. Strategies that seek to enhance insulin secretion and sensitivity are the cornerstone of diabetes treatment. Insulin biosynthesis is regulated by many physiological events; however the main driver of its secretion is circulating glucose, such that, after a meal is consumed, the levels of insulin spike in circulation. Insulin then acts on a variety of tissues in the body, including, but not limited to, adipose, liver, and muscle. The target cells are activated through the insulin receptor which then leads to increased translocation of glucose transporters to the membrane and glucose uptake. During the development of type 2 diabetes mellitus (T2DM), insulin signaling in the target tissues is impaired, and in order to overcome this resistance, the *β* cells of the pancreas begin to proliferate and produce more insulin. In cases where the pancreas is unable to produce sufficient insulin to regulate the rising glucose levels, T2DM develops. In this scenario, a variety of treatments that act to increase insulin levels or enhance insulin signaling are employed. Nevertheless, additional strategies to enhance insulin levels and signaling are of great interest in the treatment of diabetes and metabolic disease.

The investigation of hydrogen sulfide's potential involvement in glucose metabolism began in 1990 when Hayden and colleagues showed that H_2_S exposure (2.2 mM) increased circulating glucose in postpartum rats [23]. Later on, several groups began to investigate how H_2_S levels fluctuate in metabolic disease. Human studies that have examined circulating H_2_S in T2DM have found them to be reduced. Jain and colleagues found that T2DM individuals had significantly lower H_2_S compared to age matched nondiabetics [2]. Whiteman and colleagues confirmed these findings and further demonstrated that adiposity was negatively correlated with H_2_S [3]. This is of particular interest since obesity is one of the principal causes of T2DM. Unfortunately, the mechanisms driving these changes in circulating H_2_S, or their effects on glucose metabolism, were not investigated. As such, it is unclear whether the altered circulating H_2_S observed in obese individuals is a driving force in their metabolic disease. A more mechanistic understanding of how H_2_S can alter glucose metabolism has come to light through the examination of glucoregulatory hormones such as insulin and its target tissues. These pathways and their role in glucose homeostasis are described below.

## 4. H_**2**_S Production and Function in the Pancreas

The first evidence that H_2_S was produced in the pancreas and that it played a role in the regulation of insulin secretion came from Yang and colleagues. Using the INS-1 cell line, they demonstrated that *β* cells express the enzymatic machinery required to produce H_2_S, including CSE, and can produce high levels of H_2_S which blocks glucose-stimulated insulin secretion [8]. This was later confirmed in another *β* cell model, Min6 [24]. Yang and colleagues also demonstrated that treating INS-1 cells with H_2_S, or overexpressing CSE, stimulated apoptosis [7]. This latter effect appeared to be caused by increased endoplasmic reticulum stress and may be a driving factor in the reduced insulin secretion observed [7]. In addition, other groups have demonstrated the mRNA expression of both CSE and CBS in the rat pancreas and that streptozocin-induced diabetes (a model of type 1 diabetes) causes increased mRNA expression of CBS and increased H_2_S production [4]. Using a rodent model of obese diabetes (the Zucker diabetic fatty rat), Wu and colleagues demonstrated that the animals impaired glucose metabolism was due to an overproduction of pancreatic H_2_S and impaired insulin secretion [6]. Together, these studies suggest that increases in H_2_S may be responsible for a reduction in insulin secretion and ultimately the impaired glucose clearance that occurs in diabetes. However, other groups have suggested that the elevated H_2_S production from the *β* cell is occurring as a result of elevated circulating glucose and that H_2_S is acting as a pancreatic brake, which may protect these insulin producing cells from being overstimulated by chronic hyperglycemia [25]. Indeed, it was later demonstrated that mice on a high fat diet lacking CSE have significantly worse islet glucotoxicity compared to WT animals [26]. This protective role for H_2_S in *β* cell apoptosis occurs through H_2_S mediated activation of thioredoxin, a system responsible for controlling redox homeostasis that protects *β* cells from glucotoxicity. The difference in reports of the protective versus toxic effect of H_2_S in the pancreas may be due to the cell/animal model being used (whole animal versus cell studies and type 1 versus type 2 diabetes models). The differences in H_2_S concentrations used would warrant further research into what concentration threshold is protective or detrimental to cellular function. Nevertheless, H_2_S is produced in the pancreas and this appears to have important implications in insulin secretion and glucose homeostasis. How this gasotransmitter can elicit its effects on the cell is discussed below.

## 5. Mechanism of H_**2**_S Action in the Pancreas

The earliest reports on the intracellular target of H_2_S in insulin regulation were found to be an opening of the K_ATP_ channel [8]. When glucose enters the *β* cell, it generates ATP, causing the closure of ATP sensitive K_ATP_ channels and opening of calcium channels leading to depolarization and thus insulin secretion [27]. When K_ATP_ channels are kept open by H_2_S, the *β* cell is hyperpolarized and insulin secretion is suppressed. Based on this, several groups have demonstrated that compounds that suppress the production of H_2_S can increase the secretion of insulin from *β* cells [8, 24]. The precise mechanisms that cause the opening of this channel remain an active area of research. It has been suggested that direct binding of H_2_S to cysteine residues in proteins (sulfhydration) may be a potential mechanism [28]. Using the patch clamp method coupled with channel subunit mutagenesis, Jiang and colleagues demonstrated the importance of the rvKir6.1/rvSUR1 subunits in mediating K_ATP_ channel opening [29]. It should be noted however that the above studies on the precise mechanisms of H_2_S on the K_ATP_ have not been done in the *β* cell.

Voltage-dependent calcium channels (VDCCs) in the *β* cells control the movement of calcium, a crucial step in glucose-stimulated insulin release. One of the early studies examining the effect of H_2_S in *β* cells found that NaHS (an H_2_S donor) caused a decrease in the calcium oscillations caused by glucose, which ultimately led to reduced insulin secretion [24]. Using whole mouse islets, Tang and colleagues demonstrated (via patch clamp) that L-type VDCC current density is inhibited by the H_2_S donor NaHS and that islets from mice lacking CSE had reduced L-type VDCC activity [29]. Of interest, these reports of decreased VDCC activity in *β* cells and islets are in contrast to the increased calcium concentrations that result from H_2_S in cerebellar granule neurons [30]. This difference suggests that H_2_S may regulate similar intracellular pathways in distinct manners depending on the cell type.

In addition to ion channel activities, H_2_S may also regulate insulin secretion through the modulation of intracellular kinases. Several of these kinases are known to be modulated during the secretion of insulin including PI3K, ERK, AKT, and MAPK. Indeed, both endogenous and exogenous H_2_S have been shown to directly activate the p38 MAPK [7]. Importantly, activation of the MAPK/JNK pathway is a known mechanism in impaired insulin release from the *β* cell [31]. More studies are required to determine if additional cell signaling pathways are altered through the activity of H_2_S.

## 6. H_**2**_S Effects on Metabolic Tissues

The description thus far focused on the production and effects of H_2_S in the insulin secreting *β* cell. A vital part of glucose homeostasis is the function of the insulin sensitive metabolic organs, including adipose tissue, liver, and muscle.

One of the principle targets of insulin is the adipocyte. Insulin promotes the storage of excess glucose and its conversion to fat, leading to increased adiposity, a major risk factor for the development of metabolic disease. Several groups have demonstrated that adipose tissue produces H_2_S, and that gasotransmitter production and signaling in the adipocyte are altered during obesity. Feng and colleagues were the first group to describe the expression of CBS and CSE and production of H_2_S from rat adipocytes [32]. In this report they demonstrated that H_2_S impairs insulin mediated glucose uptake and that high fructose-induced diabetes led to increased production of H_2_S in epididymal adipose tissue, an effect that could be blocked by inhibiting CSE. This result points towards a negative effect of H_2_S on glucose uptake in the adipocyte. Interestingly, circulating levels of H_2_S are lower in obese humans [3], suggesting a disconnection in the increased production observed in the rodent adipose tissue. Some groups have demonstrated a positive role for H_2_S in glucose metabolism in the adipocyte. One study in 3T3L1 adipocytes found that H_2_S is required for vitamin D induced GLUT4 translocation and glucose uptake [33]. Another positive role for H_2_S in adipose tissue metabolism appears to be its role in reducing inflammatory cytokine production from resident adipose macrophages. These cytokines are a known causal factor in the development of insulin resistance in adipose and other metabolic tissues [34]. In one study, macrophages isolated from mice with diet-induced obesity produced less H_2_S and more cytokines than macrophages from lean mice [5]. Based on these reports, it may be important that future work in adipose tissue (from obese subjects) separates the adipocytes from the stromal vascular fraction. Several studies have also shown a role for the H_2_S/CSE system in perivascular adipose tissue, although most of this work has described its importance in vascular tone (reviewed in [35]) rather than glucose homeostasis.

Another key organ in the regulation of glucose metabolism is the liver. During an elevated circulating glucose scenario, insulin acts on the liver to stimulate glucose uptake and its conversion to glycogen and fatty acids for storage. In a low glucose scenario, pancreatic glucagon acts on the liver to promote the production or liberation of glucose through gluconeogenesis or glycogenolysis, respectively. Dysregulation of insulin signaling in the liver (hepatic insulin resistance) is a common phenomenon in T2DM (reviewed in [36]). The mRNA expression of both CSE and CBS was demonstrated in the liver of rats and was found to increase after inducing type 1 diabetes with STZ [4]. Later on it was demonstrated that overexpressing CSE in hepatocytes leads to reduced glycogen content. In this study, it was also shown that CSE KO animals (lower H_2_S) have a reduction in endogenous glucose production [37]. A recent study by Ju and colleagues demonstrated a mechanism by which H_2_S may directly stimulate gluconeogenesis. They found that pyruvate carboxylase (a key enzyme in gluconeogenesis) is sulfhydrated by H_2_S, which leads to increased activity and glucose production [9]. These findings seem to indicate that H_2_S production in the liver causes enhanced glucose release, an effect that could aggravate the hyperglycemia observed in diabetes. However, since type 2 diabetics are known to have lower rather than higher circulating H_2_S, further studies investigating the liver production of H_2_S during T2DM are required.

Surprisingly, there is a paucity of studies that have examined the role of H_2_S in skeletal muscle, let alone skeletal muscle glucose uptake. This may be due in part to the low or nondetectable levels of the H_2_S producing enzymes in rodent models (in contrast to the higher levels found in human muscle, reviewed in [38]). Nevertheless, future work should, at the very least, examine the effects of H_2_S donors since H_2_S may act on muscle tissue via its circulating stores.

## 7. Other Hormones and Future Work

While H_2_S plays important roles in the metabolism of hormones like insulin and glucagon, a variety of other metabolic hormones remain to be examined. One emerging area holding potential for this is the gastrointestinal endocrine system. Here, a variety of enteroendocrine cells secrete numerous peptide hormones that play important roles in glucose homeostasis and energy metabolism. Some important candidates are the insulin-stimulating incretin hormones: glucose-dependent insulinotropic polypeptide (GIP) and glucagon peptide-1 (GLP-1). Recently, Bala and colleagues examined the role of endogenous H_2_S in a GI endocrine cell line, STC-1 [39]. This cell line secretes GLP-1 and the anorexic hormone peptide YY (PYY). They found that H_2_S donors and l-cysteine impaired oleic acid-stimulated GLP-1 and PYY secretion. While their primary focus was on the modulatory effect of H_2_S on oleic acid-stimulated hormone secretion, their results support further investigation of H_2_S on GI hormone secretion and signaling. Indeed, the question remains: can GI endocrine cells produce their own H_2_S, and is the altered H_2_S level observed in obesity responsible for the dysregulation in GI hormone secretion [40]? Of importance, GLP-1 therapies have become a major tool in the treatment of type 2 diabetes [41] and recently obesity [42]. Therefore, the role H_2_S has in GLP-1 and other endocrine cells may be an additional mechanism by which this gasotransmitter can regulate glucose homeostasis.
